# Exploring the Interplay Between Vitamin D and Metabolic Parameters in Pediatric Obesity: Toward Early Biomarker Detection

**DOI:** 10.1155/jnme/4055705

**Published:** 2025-11-30

**Authors:** Valeria Calcaterra, Lucia Labati, Valentina Fabiano, Carla Colombo, Martina Loiodice, Daniele Ceruti, Gianvincenzo Zuccotti

**Affiliations:** ^1^Pediatric and Adolescent Unit, Department of Internal Medicine, University of Pavia 27100, Pavia, Italy; ^2^Pediatric Department, Buzzi Children's Hospital, Milan, Italy; ^3^Department of Biomedical and Clinical Science, University of Milan, Milan, Italy; ^4^Department of Endocrine and Metabolic Diseases, Istituto Auxologico Italiano, IRCCS, Milan, Italy; ^5^Department of Pathophysiology and Transplantation, University of Milan, Milan, Italy; ^6^Department of Biotechnology and Translational Medicine, University of Milan, Milan, Italy

**Keywords:** adiposity, children, metabolic syndrome, pediatric obesity, vitamin D

## Abstract

**Introduction:**

It is still uncertain whether vitamin D deficiency is a cause, effect, or contributing element in childhood obesity and metabolic imbalance. This study combines univariate and multivariate analyses to investigate associations between vitamin D levels, metabolic parameters, and adiposity indices, with the goal of identifying potential patterns and biomarkers among highly interrelated variables.

**Patients and Methods:**

We retrospectively analyzed data from 285 children with obesity (mean age of 10.71 ± 2.69 years). Clinical, anthropometric, metabolic, and endocrinological parameters and vitamin D levels were assessed. A control group of 55 normal-weight children, comparable in age, was included. Both univariate and multivariate statistical approaches were applied.

**Results:**

Univariate analysis revealed significant differences between weight classes in multiple clinical and biochemical parameters, including atherogenic markers, adiposity indices, and insulin-resistance indicators (*p* < 0.001 for most). In comparisons based on vitamin D status, differences were particularly marked in body mass index (BMI), insulin, triglycerides, Homeostatic Model Assessment for Insulin Resistance (HOMA-IR), and Triglyceride-Glucose (TyG) Index. Multivariate analysis identified weak but significant negative correlations between vitamin D and some clinical parameters (atherogenic coefficient, Triglyceride-Cholesterol Body Index, and HOMA-IR), with correlation coefficients ranging from −0.14 to −0.28. Principal component analysis (PCA) showed that vitamin D and BMI are often positioned on opposite axes in biplots, supporting a negative correlation. Notably, the principal component (PC) 2, dominated by BMI and vitamin D, effectively distinguished weight groups based on insulin resistance and adiposity variables. No consistent associations were observed with sex or traditional atherogenic markers.

**Conclusions:**

Vitamin D levels are significantly associated with key metabolic parameters, especially those related to insulin resistance and adiposity. Although no direct relationship was observed with classical atherogenic markers, multivariate analysis suggests possible indirect links mediated by excess adiposity. These findings support the potential role of vitamin D as a marker of metabolic health. Longitudinal studies are needed to determine whether improving vitamin D status can enhance insulin sensitivity and reduce metabolic risk in children with obesity.

## 1. Introduction

Childhood obesity has become one of the most pressing global health concerns, with its prevalence steadily increasing over recent decades [1]. According to the World Health Organization (WHO), more than 39 million children under the age of 5 years were overweight or obese in 2022, and over 340 million children and adolescents aged 5–19 years were classified as having overweight or obesity, nearly triple the number reported in 1975 [2].

In addition to well-known cardiovascular, gastrointestinal, and respiratory issues, orthopedic complications, and psychological effects, childhood obesity is strongly associated with early-onset metabolic disturbances, including insulin resistance, dyslipidemia, and hypertension [3–9]. In this context, vitamin D has emerged as a potential metabolic regulator, with functions that extend well beyond calcium homeostasis and bone health [10–13].

Recent studies have consistently shown that children with obesity are significantly more likely to have low serum levels of vitamin D compared to their normal-weight peers [14–18]. Several mechanisms may explain this deficiency, including sequestration of vitamin D in adipose tissue, which reduces its bioavailability [19–22]; reduced sun exposure due to sedentary lifestyles; and poor dietary intake of vitamin D-rich foods, such as fatty fish, fortified milk, and eggs [19–22].

Beyond its classical role in calcium and bone homeostasis, vitamin D exerts significant effects on various metabolic pathways [23–26]. Its active form, calcitriol (1,25-dihydroxyvitamin D), binds to vitamin D receptors (VDR) expressed in multiple tissues, including the pancreas, liver, adipose tissue, and skeletal muscle, indicating a broader regulatory role in energy metabolism, glucose homeostasis, and lipid regulation [27–29].

Metabolically, low vitamin D levels in children with obesity have been associated with increased insulin resistance [30, 31] and a higher risk of developing type 2 diabetes during adolescence [10]. Additionally, altered lipid profiles, including elevated triglycerides (TGs), total cholesterol (TC), and LDL cholesterol, have been observed, along with increased levels of inflammatory markers, such as C-reactive protein [30, 31].

Despite these associations, it remains unclear whether vitamin D deficiency is a cause, consequence, or contributing factor in the development of metabolic dysregulation in children with obesity. However, gaining a deeper understanding of this relationship may lead to more targeted intervention strategies, including the use of vitamin D supplementation as an adjunct in the management of pediatric obesity and its metabolic consequences.

Multivariate pattern analysis is a widely used tool in biomedical research, particularly in disease diagnosis and treatment, due to its ability to account for complex intervariable correlations and uncover meaningful associations within the data [32, 33]. Compared to univariate approaches, which evaluate each variable in isolation, multivariate methods provide a more integrated perspective, capturing interactions and patterns that may otherwise remain undetected.

In this study, we combined univariate and multivariate approaches to explore the associations between vitamin D levels, metabolic parameters, and adiposity indices, aiming to identify meaningful patterns and potential biomarkers among highly interrelated variables. We included children with both overweight and obesity, along with a control group of normal-weight peers, to enable the investigation of the progressive influence of weight status on vitamin D levels and related metabolic parameters. Early detection of these markers may be crucial for the effective management of patients with obesity, as well as for the prevention and treatment of cardiometabolic diseases from an early age.

## 2. Patients and Methods

### 2.1. Study Population

A cohort of 285 Caucasian children and adolescents (140 boys and 145 girls), aged between 6 and 18 years (mean age of 10.71 ± 2.69), diagnosed with overweight or obesity, was retrospectively recruited from the Pediatric Endocrinology Outpatient Clinic at Buzzi Children's Hospital in Milan between October 2020 and June 2023.

Eligibility criteria required a body mass index (BMI) at or above the 75th percentile for age and sex. Informed consent had to be provided by a parent or legal guardian. Exclusion criteria included the presence of secondary forms of obesity; use of vitamin D supplements within the past 6 months; any ongoing medication; hormonal disorders, such as hypothyroidism, hyperthyroidism, Cushing's syndrome, and growth hormone deficiencies; chronic conditions including renal or hepatic insufficiency, malabsorption syndromes (e.g., celiac disease or Crohn's disease), and autoimmune disorders; and any acute illnesses.

A control group composed of 55 normal-weight children, matched by age and sex, was also included. These children had no known history of vitamin D deficiency, had not received any vitamin D supplementation in the previous 6 months, and did not present with any acute or chronic clinical conditions that could potentially affect serum vitamin D levels or its biological functionality.

Both groups underwent comprehensive clinical assessments and blood testing for biochemical analysis.

The study received ethical approval from the Institutional Ethics Committee (Protocol ID: 2020/ST/234 MI) and was conducted in accordance with international Good Clinical Practice (GCP) guidelines and applicable regulatory standards. All participants, or their legal guardians, provided written informed consent.

### 2.2. Methods

#### 2.2.1. Clinical Evaluation

All participants were evaluated during outpatient visits that included physical assessments and venous blood sampling.

Anthropometric data, such as body weight, height, BMI, waist circumference (WC), and blood pressure, were measured using standardized techniques. Measurements were taken with participants barefoot and dressed in underwear. Weight was measured using a digital scale accurate to ±100 g, and height was assessed with a wall-mounted stadiometer (±1 mm accuracy). WC was measured using a nonelastic tape placed horizontally between the lower rib margin and the iliac crest.

BMI was calculated as weight (kg) divided by height squared (m^2^), and *z*-scores were determined using WHO reference standards [34]. Overweight was defined as a BMI-for-age *z*-score > +1 standard deviation (SD) and obesity as a *z*-score > +2 SD.

In addition to BMI, BMI-z score, and WC, adiposity indexes, including waist-to-height ratio (WHtR), A Body Shape Index (ABSI), Tri-Ponderal Mass Index (TMI), Visceral Adiposity Index (VAI), and Conicity Index (ConI), were considered and calculated as follows:

• WHtR = WC/Ht
• ABSI = 1000 × WC × Wt^−2/3^ × Ht^5/6^ [35]
• TMI = weight (kg)/height (m)^3^ [36]
• ConI = WC/(0.109 × (Wt/Ht)^0.5^) [37].
• VAI [38].

For males: WC/(39.68 + 1.88 × BMI)WC/(39.68 + 1.88 × BMI)WC/(39.68 + 1.88 × BMI) × (TG/1.03) × (1.31/HDL-C).

For females: WC/(36.58 + 1.89 × BMI)WC/(36.58 + 1.89 × BMI)WC/(36.58 + 1.89 × BMI) × (TG/0.81) × (1.52/HDL-C).

Pubertal development was evaluated during the clinical assessment using Tanner staging. Participants were categorized into three groups: Stage 1 (prepubertal), Stages 2–3 (early pubertal), and Stages 4–5 (late pubertal to adult) [39].

#### 2.2.2. Biochemical Evaluation

Venous blood was drawn following an overnight fast of at least 8 h. The analyses focused on vitamin D levels, lipid profile, glucose metabolism, and insulin sensitivity markers.

Vitamin D status was assessed by measuring 25-hydroxyvitamin D (25-OH-D) levels using the cobas 6000 analyzer (modules c501 and e601, Roche Diagnostics GmbH, Mannheim, Germany). Deficiency was defined as 25-OH-D < 20 ng/mL [30].

The lipid panel included TC, high-density lipoprotein cholesterol (HDL-C), low-density lipoprotein cholesterol (LDL-C), and TGs, all measured on the same analyzer. Derived lipid ratios were also calculated using the following formula:

− TG/HDL-C [11, 40];
− Atherogenic Index of Plasma (AIP): log[TG/HDL-C]) [11, 40];
− Cardiometabolic Risk Index I (CRI-I): TC/HDL-C [11, 40];
− Cardiometabolic Risk Index II (CRI-II): LDL-C/HDL-C [11, 40];
− Atherogenic coefficient (AC) = (TC–HDL)/HDL [11, 40];
− Triglyceride-Cholesterol Body Index (TCBI): [TG (mg/dL) × TC (mg/dL) × body weight (kg)]/1000 [11, 40].

We chose to include derived lipid ratios because they provide enhanced insight into cardiometabolic risk beyond individual lipid measurements. These ratios reflect the balance between atherogenic (harmful) and protective (anti-atherogenic) lipoproteins, serving as integrative markers of overall cardiovascular health.

To evaluate glucose metabolism, fasting insulin, glucose, and glycated hemoglobin (HbA1c) were measured using the cobas 6000 system. To provide a more comprehensive assessment of insulin resistance beyond individual measures, we included both traditional and composite indices. These markers included the following:

− Homeostatic Model Assessment for Insulin Resistance (HOMA-IR): (fasting glucose in mg/dL × fasting insulin in μU/mL)/405 [41].
− Triglyceride-Glucose Index (TyG)**:** calculated as logTG(mg/dL) × glucose(mg/dL)/2TG (mg/dL) × glucose (mg/dL)/2TG(mg/dL) × glucose(mg/dL)/2 [42].
− TyG-BMI = TyG index *x* BMI [42].
− TyG-WC = [(TyG index) × (WC (cm)] [42].
− TyG-WHtR = (TyG index) × WHtR [42],
− These combined indices are considered superior to single parameters alone, as they integrate both metabolic and anthropometric risk components, thereby improving early identification of insulin-resistance and associated metabolic dysfunctions [42–45].

## 3. Statistical Analysis

First, a univariate statistical analysis was performed to examine the characteristics of the entire sample in terms of mean and SD, which was then divided into three groups, according to the weight class criterion and then into two groups based on the vitamin D level. This first phase of analysis allowed us to evaluate whether significant differences emerged between the various groups for the individual variables under examination. In particular, to compare the three subgroups divided by weight classes, one-way ANOVA tests were performed, whereas comparisons between the two groups with pathological and nonpathological vitamin D levels were conducted using independent-samples *t*-tests.

To identify trends in the distributions of the variables and estimate the probability density function of the analyzed variables, a nonparametric kernel density estimation method was applied. The function was represented by violin plots, which include a scatter plot and a box plot showing the median and first and third quartile values, to obtain a complete view of the distribution. The plots of the atherogenic markers were analyzed and compared by grouping the subjects by sex, weight classes, and vitamin D levels.

Furthermore, to identify a measure of linear correlation between each pair of variables, Pearson correlation coefficients were calculated and represented in a matrix correlation graph.

Subsequently, multiple linear regression models were built to see what relationships exist between the various parameters (independent variables) and vitamin D, thought of as the dependent variable. The *p*-values for the individual associations in the regression models were then calculated.

After standardizing the values detected for the variables under examination to make them more homogeneous and comparable, an overall *z*-score was also calculated by adding the individual scores. This was then related to the vitamin D levels through the calculation of the *p*-value, the Pearson correlation coefficient, and the construction of some scatter plots.

In addition, to further investigate the potential associations between the parameters studied with vitamin D and the subjects' BMI, a principal component analysis (PCA) was performed. This method allows to reduce the dimensionality of the dataset by identifying the principal components (PCs) (up to three), while preserving the maximum variability of the data. In this way, a new multivariate space is created, defined by the directions of the PCs, each of which is characterized by a percentage of explained variance of the data reported in the scree plot. The standardized component scores of the individual subjects were then represented in the biplots: In these graphs, the variables present in the dataset were represented through their projection as vectors (black arrows labeled) on the subspace defined by the new axes. This type of representation allows us to explore the correlations among the structures present in the data. If two variables are strongly related, the two vectors that represent them are projected close, almost parallel (in this case, the correlation is positive), while in the opposite situation, the arrows are close to the antiparallelism condition (the correlation is negative). On the contrary, if there are no particular correlations, the two vectors will form a 90-degree angle. Once the PCs were extracted, within the three parameter groupings (atherogenic markers, insulin-resistance profile parameters, and adiposity indices), the various PC relationships with BMI and vitamin D were investigated through the calculation of *p*-values and Pearson coefficient.

All statistical analyses were conducted with Microsoft Excel software with the addition of some add-ons and extensions of the program, such as Real Statistics, which made it possible to conduct a more accurate and complete analysis, especially with regard to multivariate analysis and the study of correlations with PCs.

## 4. Results

### 4.1. Univariate Analysis

The Table 1 shows the clinical characteristics, atherogenic markers, insulin-resistance parameters, and adiposity indices of the patients, divided into weight classes and by the presence or absence of vitamin D deficiency.

The largest differences between children with normal weight, overweight, and obesity emerged for the variables: weight (*p* < 0.001), CV (*p* < 0.001), CV/H (*p* < 0.001), BMI (*p* < 0.001), glycemia (*p* < 0.001), insulin (*p* < 0.001), HbA1c (*p* < 0.001), HDL (*p* = 0.004), TG (*p* < 0.001), TG/HDL (*p* < 0.001), AIP (*p* < 0.001), CRI-I (*p* < 0.001), CRI-II (*p* < 0.001), TCBI (*p* < 0.001), HOMA-IR (*p* < 0.001), TyG (*p* < 0.001), TyG-BMI (*p* < 0.001), TyG-WC (*p* = 0.030), and TyG-WHtR (*p* < 0.001). For vitamin D, the *p*-value obtained was not significant (*p* = 0.075).

As regards the comparison between subjects with normal or pathological vitamin D levels, discrepancies emerged mainly between the following variables: age (*p* = 0.043), weight (*p* = 0.007), BMI (*p* < 0.001), insulin (*p* = 0.001), TGs (*p* = 0.05), TG/HDL (*p* = 0.05), AIP (*p* = 0.05), AC (*p* = 0.03), TCBI (*p* = 0.017), HOMA-IR (*p* = 0.001), TyG (*p* = 0.031), TyG-BMI (*p* = 0.005), TyG-WC (*p* = 0.05), and TMI (*p* = 0.037).

**Figure 1:**
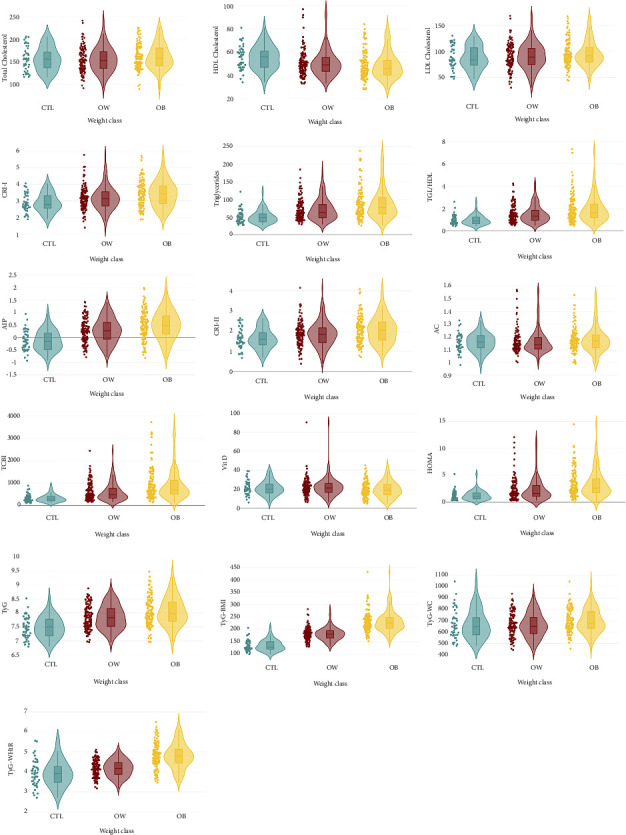
Atherogenic markers, insulin resistance, and vitamin D**:** kernel density estimation by violin plots. Patients were divided by weight class: in green, the distribution of observations in normal weight; in red, overweight; and in yellow, the distribution of observations in obesity.

Violin plots were drawn up for the three groups of parameters: atherogenic markers, insulin resistance, and adiposity indices, dividing the sample according to three different criteria (sex, weight class, and vitamin D level), see Supporting Information: figure S1 (violin plot for atherogenic markers with subjects divided by sex), figure S2 (violin plot for atherogenic markers with subjects divided by vitamin D level), and figure S3 (biplot graphs for atherogenic markers with comparison by sex). Only the atherogenic markers and insulin-resistance parameters are reported below in Figure 1, with the subjects divided into normal weight (CTL), overweight (OW), and obesity (OB). All other data emerging from the analysis and comparisons are summarized in written form, without graphic support. The violin plots, including the scatter diagram and the inner box plot, show the differences in the distributions. In the comparison between males and females, some differences in the shape of the graph were found especially in TGs, TG/HDL ratio, AC, TCBI, and vitamin D. As regards the comparison among weight classes, the OB group showed a more widely dispersed distribution of the variables related to atherogenic markers compared to normal weight and overweight. In particular, differences were found in biochemical variables, such as TGs, TG/HDL, AIP, CRI-I, CRI-II, and TCBI, an inverse trend instead for HDL cholesterol which was more distributed in normal weight, while no significant differences were observed for TC, LDL, and vitamin D.

Even in the comparison between the violin graphs relating to the insulin-resistance parameters, a more relaxed distribution emerges in the OB group, followed by the overweight and the normal weight, except for the TyG-WC in which the three diagrams are very similar. For HOMA-IR, TyG, TyG-BMI, and TyG-WHtR in the obese, values are also recorded that are included in a globally higher range.

Finally, in the comparison of violin plots for vitamin D levels, some differences were found in the dispersion of fasting TGs, TG/HDL ratio, and TCBI index, with patients with pathological vitamin D level (value less than 20 mg/dL) showing a wider distribution than those with normal value.

From the analysis of the correlogram reported in Table 2, no significant associations emerged between vitamin D and the group of metabolic marker variables (either among atherogenic markers or among insulin-resistance indices).

### 4.2. Multivariate Analysis

The first step of the multivariate analysis consisted in the construction of a multivariate linear regression model aimed at identifying any correlations between the variables of the three groups with vitamin D. In particular, statistically significant correlations emerged and were weighted through the calculation of *p*-values, with the parameters AC (*p* = 0.014), TCBI (*p* = 0.051), HOMA-IR (*p* = 0.011), and VAI (*p* = 0.063), Table 3.

Subsequently, after the standardization of the dataset values for the various variables (subtracting the mean and dividing by the SD), an overall z-score was calculated for each subject. It was then assessed whether there were associations between these scores and vitamin D values through the production of scatter plots and the calculation of Pearson correlation coefficients (*r* = −0.19 for atherogenic markers, *r* = −0.28 for insulin-resistance markers, and *r* = −0.14 for adiposity index).

Following these first analyses from the multivariate approach, as pairwise associations do not take into account joint relationships with other variables, an in-depth analysis of the association structure between parameters was conducted using PCA. Three separate analyses were performed, each for the three groups of variables previously assessed: Vitamin D and BMI were included in each group to analyze whether there were intrinsic relationships between these and the other variables.

**Figure 2:**
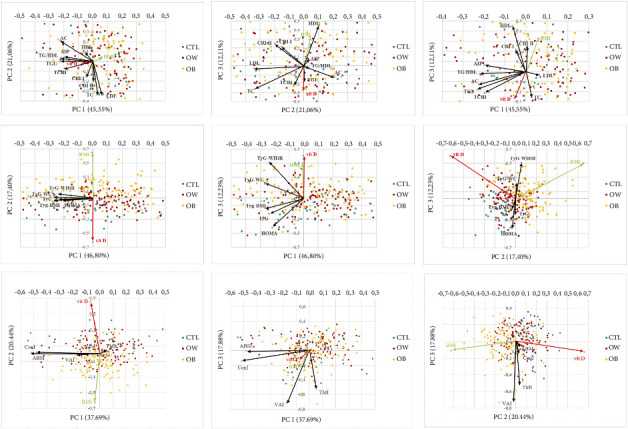
Principal component analysis (PCA) biplot graphs with standardized score components of the study participants based on clinical and laboratory variables. Each individual point represents one of the subjects of the cohort colored by group of weight class (blue = controls, red = overweight, and yellow = obesity). The three graphs in the first column show the two main planes PC1 and PC2 extracted from the analysis; the second column shows PC1 and PC3, while the three graphs in the third column show PC2 and PC3 on the two Cartesian axes. In the labels presented, the percentages of variance explained from each component. The three graphs in the first row show the atherogenic markers, the insulin resistance indices in the three figures in the central row, and the adiposity indices in the three images at the bottom.

In the first group of atherogenic markers, the first three PCs explained 78.72% of the variance in the data (PC1 = 45.55%, PC2 = 21.06%, and PC3 = 12.11%), as presented in the scree plot table. The projections of the original variables onto the three planes are shown in the following figure. Two sets of biplots were produced, one in which the component scores were divided by sex (the observations derived from female subjects are in pink, while those of males are in blue) and one by weight class (normal weight in blue, overweight in red, and obese subjects in yellow). The first axis (PC1) was characterized mainly by the variables TGs, TG/HDL ratio, AIP, AC, and TCBI. The second axis (PC2) was characterized by the variables TC, LDL, CRI-I, and CRI-II. The third axis (PC3) was characterized by the variables HDL, CRI-I, CRI-II, vitamin D, and BMI. In Figure 2 and Table 4, biplot analysis and the summary of PCA, including explained variance metrics, were reported.

From the biplot analysis, it can be seen that in all the planes defined by the PCs, it was found that vitamin D and BMI are represented by two diametrically opposed vectors (Figure 2). In the PC1-PC2 plane, the variables HDL, AC, TG, TG/HDL ratio, TCBI, and vitamin D were positively correlated and grouped as well as the variables CRI-I, CRI-II, TC, and LDL, and this last group shows no relationship with BMI and vitamin D. As regards the PC1-PC3 plane, particular relationships were highlighted between BMI, HDL, and AIP, and between vitamin D and TGs, while there was almost no association between vitamin D and CRI-I, II, TG/HDL, and AC. Finally, in the PC2-PC3 plane, relationships emerged between AIP, TG/HDL, AC, TG, and TCBI and between CRI-I, CRI-II, and HDL with BMI. Perfect perpendicularity, therefore, no relationships were found between BMI and vitamin D, neither with LDL nor with AIP.

In the PCA performed among the variables belonging to the group of insulin-resistance parameters, the first three PCs identified explained 76.43% of the variance in the data (PC1 = 46.80%, PC2 = 17.40%, and PC3 = 12.23%). Also, in this case, two sets of biplots were produced, according to gender and weight class. The first axis (PC1) was mainly characterized by the variables TyG, TyG-BMI, TyG-WC, and TyG-WHtR. Vitamin D and BMI variables characterized mainly the second axis (PC2), whereas the third axis (PC3) was characterized by the variables vitamin D, BMI, TyG-WHtR, and HOMA-IR.

Looking at the biplot graphs, the following considerations emerge: In the PC1-PC2 plane, all the insulin-resistance parameters TyG, TyG-BMI, TyG-WC, HOMA-IR, and TyG-WHtR were found to be strongly positively correlated and grouped, while they are almost perpendicular; therefore, they do not highlight any relationship with either BMI or vitamin D. As regards the PC1-PC3 plane, particular relationships were highlighted between TyG and TyG-BMI, while no association was found between vitamin D and BMI and TyG-WC. Finally, in the PC2-PC3 plane, strong relationships emerged between TyG-WC and TyG-WHtR and between TyG-BMI, HOMA-IR, and TyG. Perfect perpendicularity, therefore, no relationships were found between BMI and vitamin D. In the PC1-PC3 plane, unlike what was seen for the atherogenic markers, a very strong relationship emerges between BMI and vitamin D. Diametrically opposite positions, therefore, complete negative correlation between these two variables in the PC1-PC2 plane.

As a final multivariate PCA analysis, we attempted to extrapolate the PCs between the adiposity indices. The first three PCs identified explained 76.00% of the variance in the data (PC1 = 37.69%, PC2 = 20.44%, and PC3 = 17.88%). Previously, two sets of biplots were represented, based on gender and weight class. The first axis (PC1) was mainly characterized by the variables ABSI, ConI, and VAI. The variables vitamin D and BMI represented the second axis (PC2), while the third axis (PC3) was characterized by the variables TMI and VAI.

From the analysis of these biplots, it can be seen that both in the PC1-PC2 plane and in the PC2-PC3 plane, BMI and vitamin D are represented by almost diametrically opposed vectors; therefore, this highlights a strongly negative correlation. On the contrary, in the PC1-PC3 plane, a highly positive correlation is shown between the two variables BMI and vitamin D. Other positive correlations emerged between the variables ABSI, ConI, and VAI in the PC1-PC2 plane where, however, these are perpendicular to both BMI and vitamin D. Another strong positive correlation emerged between VAI, TMI, and ConI in the PC2-PC3 plane; also, in this case, there was no correlation with BMI and vitamin D. In the PC1-PC3 plane, correlations emerge, although of moderate intensity, between vitamin D and BMI with VAI and ConI.

From a comparative analysis of the various biplot graphs, considering the plane defined by PC1 and PC2 and that by PC2 and PC3, it emerges that, for the insulin-resistance parameters, the observations of the subjects divided by weight classes are grouped, while in the plane where the main components PC1 and PC3 appear, the observations are mixed and more dispersed. This highlights the fact that PC2, characterized by vitamin D and BMI, is crucial in differentiating the three groups for the insulin-resistance parameters.

The same type of considerations made previously also emerge from the comparison between the biplots linked to the main components of the adiposity indices: Also, in this case, the observations of the subjects divided into normal weight, overweight, and obesity differ more clearly in the two planes in which PC2 is present, an axis strongly characterized by the BMI and vitamin D variables.

However, no particular correlations were noted in the distinction between the sexes (the observations were always quite mixed and not well directed according to a main direction) nor with regard to the parallel between the biplots constructed with the planes of the atherogenic markers.

## 5. Discussion

The overall analysis, which combined univariate and multivariate approaches, revealed a significant association between vitamin D levels and several metabolic parameters, particularly those related to insulin resistance and adiposity. Even though no direct association was found with traditional atherogenic markers, PCA suggested that indirect or more complex interactions may exist, potentially mediated by adiposity and/or insulin resistance.

Obesity in children is becoming an increasingly urgent matter on the global health agenda [1] and is frequently associated with nutritional disturbances, including vitamin D deficiency [14–19]. Vitamin D plays an important role not only in bone health but also in metabolic and cardiovascular morbidities commonly associated with childhood obesity [23], particularly in glucose metabolism and insulin sensitivity. The active form of vitamin D, calcitriol (1.25(OH)_2_D), binds to VDR found in pancreatic *β*-cells and insulin-responsive tissues, such as skeletal muscle and adipose tissue. This interaction is thought to enhance insulin secretion and improve insulin sensitivity by modulating intracellular calcium levels and inflammatory responses [46].

In pediatric populations, several studies have reported an inverse relationship between serum vitamin D levels and markers of insulin resistance, such as fasting insulin and HOMA-IR [47–49]. Children with vitamin D deficiency are more likely to exhibit impaired glucose tolerance, higher fasting insulin levels, and increased risk for metabolic syndrome [50]. Moreover, the TyG index has also been found to correlate negatively with vitamin D status in children and adolescents [51]. Vitamin D deficiency is linked to insulin resistance through several mechanisms. It reduces insulin sensitivity by impairing insulin receptor function, increases inflammation by elevating inflammatory cytokines, and disrupts calcium regulation, affecting insulin secretion. Additionally, vitamin D deficiency leads to dyslipidemia, increased visceral fat, and impaired pancreatic function, all of which contribute to insulin resistance [52–56].

According to the literature [17, 57], across all multivariate analyses, particularly in PCA, we confirmed a consistent and clear inverse relationship between vitamin D levels and weight excess. This association was especially strong within groups of insulin-resistance markers and adiposity parameters, as shown by significant correlations with HOMA-IR and TyG-derived indices, and a more limited association with VAI. Thus, these findings support a link between vitamin D deficiency and impaired glycemic metabolic function and insulin sensitivity.

In recent years, increasing attention has also been given to the relationship between serum vitamin D levels and lipid metabolism in children and adolescents. Several studies have reported that low levels of 25(OH)D are associated with an unfavorable lipid profile, including higher TG, elevated TC and LDL-C, and lower HDL-C [50, 58]. However, others have failed to find significant independent associations after adjusting for BMI or insulin sensitivity [50, 59].

In our study, neither univariate nor multivariate analysis revealed a significant direct correlation between vitamin D and major atherogenic markers (such as TC, LDL, CRI-I, CRI-II, and AIP). However, PCA suggested that indirect or more complex interactions may exist, potentially mediated by adiposity or insulin resistance. These results confirm that vitamin D may not exert a direct lipid-lowering effect, but rather that its influence on the lipid profile is indirect and mediated through its broader roles in adipose tissue function, inflammation, and insulin signaling. Several mechanistic pathways support this interpretation. For instance, vitamin D has been shown to regulate lipoprotein lipase (LPL) activity, an enzyme crucial for the hydrolysis of TG-rich lipoproteins. Additionally, its anti-inflammatory properties may help modulate lipid metabolism by reducing cytokine-driven hepatic lipogenesis and improving endothelial function, both of which are involved in lipid transport and vascular health [59]. These pathways may help explain the absence of a significant direct correlation in traditional analyses, while multivariate approaches, such as PCA, are more sensitive to capturing these complex, interconnected effects.

The literature supports the idea that gender-related variations may exist in the metabolic effects of vitamin D during childhood and adolescence, potentially influenced by hormonal, behavioral, and physiological factors. Several cross-sectional investigations have reported that female children and adolescents tend to have lower serum 25(OH)D levels compared to their male counterparts. This difference has been attributed to a combination of factors, including higher body fat percentage in females, reduced sun exposure, and hormonal changes occurring during puberty [18]. In terms of metabolic outcomes, certain studies have observed sex-specific patterns in the association between vitamin D and key metabolic parameters, such as lipid profile and insulin resistance. For instance, Jang et al. [60] reported that low vitamin D levels were more significantly associated with higher blood glucose and insulin resistance overall metabolic risk in females, suggesting a possible sex-dependent metabolic vulnerability linked to vitamin D status.

Despite some findings indicating potential sex-based differences, not all studies confirm such variations. In many cases, the associations between vitamin D and metabolic parameters lose statistical significance after adjusting for confounding variables, such as age, BMI, and pubertal stage. In our study, although some differences in individual metabolic parameters were detected, the distribution of variables did not show systematic differences based on sex, supporting the notion that the observed associations between vitamin D and metabolic parameters are likely independent of gender [29, 61, 62]. This suggests that, while sex may act as a modifying factor, it is unlikely to be a primary determinant of the relationship between vitamin D and metabolic health in pediatric populations.

This study has several limitations. First, the relatively small sample size, especially in the control group, warrants caution in interpreting the findings and limits the generalizability of the results. Although the control group is smaller than the treatment group, and this imbalance may affect statistical power, PCA was applied to reduce dimensionality and identify underlying patterns that could distinguish between the two groups. Further research with larger cohorts is needed to validate these observations. Another limitation is the absence of detailed lifestyle information, such as dietary habits and physical activity, which may influence vitamin D status and confound metabolic outcomes. Moreover, a lack of data on family history of diabetes and/or dyslipidemia and early-onset cardiovascular disease prevented risk stratification based on genetic predisposition. Finally, longitudinal and interventional studies will be essential to confirm these results and clarify the underlying mechanisms linking vitamin D status with metabolic parameters in children.

## 6. Conclusions

Our findings suggest that vitamin D deficiency is associated with a more compromised metabolic profile, particularly in individuals with excess weight. Vitamin D appears to be a potential indicator of metabolic health, more closely linked to adiposity and insulin resistance than to traditional atherogenic risk factors. Vitamin D status could be integrated into multifactorial prevention models, particularly for identifying high-risk individuals early, before overt metabolic syndrome or diabetes develops. Future longitudinal studies should aim to evaluate whether improving vitamin D levels through supplementation or lifestyle modification can positively influence insulin sensitivity and other metabolic outcomes over time.

## Figures and Tables

**Table 1:** Clinical features, atherogenic markers, insulin resistance parameters, and adiposity indices of the patients included in the analysis.

	Total (*n* = 285)	Control (*n* = 47)	Overweight (*n* = 119)	Obesity (*n* = 119)	Vitamin D < 20 mg/dL (*n* = 137)	Vitamin D ≥ 20 mg/dL (*n* = 148)
Age (yrs)	10.84 ± 2.56	11.25 ± 2.14	10.86 ± 2.30	10.65 ± 2.92	11.15 ± 2.76	10.55 ± 2.33
Weight (kg)	53.79 ± 18.18	39.4 ± 10.14	50.69 ± 13.83	62.58 ± 19.84	56.95 ± 19.42	50.87 ± 16.47
Height (m)	1.46 ± 0.14	1.46 ± 0.12	1.47 ± 0.14	1.46 ± 0.15	1.48 ± 0.15	1.45 ± 0.14
WC (cm)	81.19 ± 11.48	76.46 ± 12.83	77.63 ± 8.77	86.63 ± 11.18	82.17 ± 12.03	80.29 ± 10.91
WHtR	0.56 ± 0.07	0.53 ± 0.09	0.53 ± 0.05	0.60 ± 0.07	0.56 ± 0.08	0.56 ± 0.07
Vitamin D (mg/dL)	21.07 ± 8.97	21.30 ± 7.44	22.34 ± 9.79	19.70 ± 8.53	14.38 ± 3.99	27.26 ± 7.78
BMI (Kg/m^2)^	24.55 ± 5.11	18.15 ± 2.55	23.07 ± 2.67	28.56 ± 4.29	25.46 ± 5.59	23.70 ± 4.47
Glycemia (mg/dL)	78.78 ± 10.93	73.91 ± 7.82	78.26 ± 10.57	81.22 ± 11.68	79.92 ± 12.03	77.72 ± 9.74
Insulin (μU/mL)	12.90 ± 10.32	6.64 ± 4.27	11.81 ± 10.42	16.50 ± 10.59	15.02 ± 11.65	10.95 ± 8.54
Hb1Ac (%)	5.26 ± 0.44	5.92 ± 3.02	5.22 ± 1.19	5.18 ± 1.41	5.24 ± 1.96	5.27 ± 1.87
SBP (mmHg)	104.78 ± 14.11	101.13 ± 8.68	104.27 ± 23.3	106.89 ± 33.41	106.35 ± 27.33	103.33 ± 25.80
DBP (mmHg)	64.43 ± 10.29	62.53 ± 9.16	64.22 ± 15.13	65.47 ± 21.79	65.22 ± 18.36	63.70 ± 16.81
Total cholesterol	158.12 ± 27.32	156.51 ± 26.02	155.61 ± 27.54	161.27 ± 27.50	159.36 ± 27.66	156.97 ± 27.04
HDL	50.73 ± 11.53	55.37 ± 10.14	50.78 ± 11.39	48.84 ± 11.75	50.06 ± 11.69	51.35 ± 11.39
LDL	92.64 ± 24.32	88.07 ± 22.13	90.72 ± 24.38	96.37 ± 24.73	93.56 ± 25.18	91.79 ± 23.55
TG	74.98 ± 37.34	51.06 ± 18.81	71.64 ± 30.14	87.77 ± 43.66	79.82 ± 39.37	70.50 ± 34.90
TG/HDL	1.60 ± 1.01	0.96 ± 0.43	1.50 ± 0.78	1.96 ± 1.22	1.73 ± 1.06	1.49 ± 0.95
AIP	0.31 ± 0.56	−0.12 ± 0.41	0.29 ± 0.49	0.51 ± 0.58	0.38 ± 0.58	0.25 ± 0.53
CRI-I	3.23 ± 0.74	2.89 ± 0.54	3.16 ± 0.71	3.43 ± 0.79	3.31 ± 0.80	3.16 ± 0.68
CRI-II	1.92 ± 0.64	1.65 ± 0.51	1.87 ± 0.62	2.07 ± 0.67	1.97 ± 0.69	1.87 ± 0.59
AC	1.17 ± 0.10	1.16 ± 0.07	1.17 ± 0.10	1.18 ± 0.10	1.18 ± 0.10	1.16 ± 0.09
TCBI	693.29 ± 571.71	321.53 ± 170.54	598.66 ± 398.59	934.74 ± 702.56	787.85 ± 630.06	605.76 ± 498.16
HOMA-IR	2.61 ± 2.30	1.25 ± 0.89	2.34 ± 2.16	3.41 ± 2.51	3.09 ± 2.64	2.17 ± 1.83
TyG	7.87 ± 0.50	7.48 ± 0.38	7.84 ± 0.42	8.06 ± 0.51	7.94 ± 0.52	7.81 ± 0.47
Tyg-BMI	194.53 ± 47.91	135.93 ± 22.11	181.32 ± 25.99	934.74 ± 702.56	787.85 ± 630.06	605.76 ± 498.16
TyG-WC	639.63 ± 104.86	321.53 ± 170.54	598.66 ± 398.59	230.89 ± 42.20	203.62 ± 52.25	186.12 ± 41.96
TyG-WHtR	4.39 ± 0.66	3.92 ± 0.65	4.16 ± 0.41	4.80 ± 0.64	4.43 ± 0.72	4.35 ± 0.60
ABSI	0.08 ± 0.01	0.09 ± 0.02	0.08 ± 0.01	0.08 ± 0.01	0.08 ± 0.01	0.08 ± 0.01
TMI	16.80 ± 3.19	12.45 ± 1.58	15.83 ± 1.61	19.49 ± 2.29	17.22 ± 3.48	16.42 ± 2.86
ConI	1.26 ± 0.16	1.37 ± 0.25	1.23 ± 0.11	1.24 ± 0.13	1.25 ± 0.17	1.27 ± 0.15
VAI	2.42 ± 1.56	1.55 ± 0.71	2.31 ± 1.39	2.88 ± 1.80	2.61 ± 1.77	2.25 ± 1.33

*Note:* TyG, Triglyceride-Glucose Index; WHtR, waist-to-height ratio; ABSI, Body Shape Index; TMI, Tri-Ponderal Mass Index; ConI, Conicity Index.

Abbreviations: AC, atherogenic coefficient; AIP, Atherogenic Index of Plasma; CRI-I, Cardiometabolic Risk Index I; CRI-II, Cardiometabolic Risk Index II; DBP, diastolic blood pressure; SBP, systolic blood pressure; TC, Total cholesterol; TCBI, Triglyceride-Cholesterol Body Index; TG, triglycerides; VAI, Visceral Adiposity Index.

**Table 2:** Correlogram for metabolic markers and vitamin D.

	TC	HDL	LDL	TG	TG/HDL	AIP	CRI-I	CRI-II	AC	TCBI	Vitamin D	HOMA-IR	TyG	Tyg-BMI	TyG-WC	TyG-WHtR
TC	1.00	−0.30	0.90	0.29	0.13	0.11	0.46	0.50	−0.20	0.40	−0.10	0.12	0.31	0.14	0.19	0.24
HDL		1.00	−0.05	−0.33	−0.55	−0.66	−0.67	−0.62	−0.24	−0.26	0.03	−0.18	−0.31	−0.30	−0.24	−0.13
LDL			1.00	0.25	0.19	0.23	0.69	0.78	−0.38	0.37	−0.08	0.14	0.29	0.17	0.22	0.25
TG				1.00	0.94	0.89	0.54	0.40	0.56	0.87	−0.19	0.42	0.92	0.62	0.49	0.44
TG/HDL					1.00	0.93	0.66	0.51	0.56	0.83	−0.18	0.41	0.86	0.63	0.48	0.40
AIP						1.00	0.70	0.58	0.52	0.75	−0.16	0.42	0.89	0.63	0.50	0.41
CRI-I							1.00	0.97	0.08	0.58	−0.13	0.28	0.54	0.43	0.39	0.32
CRI-II								1.00	−0.15	0.47	−0.10	0.23	0.42	0.35	0.33	0.29
AC									1.00	0.39	−0.14	0.16	0.48	0.29	0.23	0.14
TCBI										1.00	−0.22	0.52	0.80	0.76	0.61	0.39
Vitamin D											1.00	−0.27	−0.20	−0.24	−0.23	−0.19
HOMA-IR												1.00	0.54	0.58	0.50	0.33
TyG													1.00	0.67	0.53	0.47
Tyg-BMI														1.00	0.70	0.54
TyG-WC															1.00	0.80
TyG-WHtR																1.00

*Note:* WHtR, waist-to-height ratio.

Abbreviations: AC, atherogenic coefficient; AIP, Atherogenic Index of Plasma; CRI-I, Cardiometabolic Risk Index I; CRI-II, Cardiometabolic Risk Index II; TC, Total cholesterol; TCBI, Triglyceride-Cholesterol Body Index; TG, triglycerides.

**Table 3:** Multivariate linear regression model results.

Parameter	*p*-value
Atherogenic markers	
Total cholesterol	0.612
HDL	0.443
LDL	0.631
TG	0.726
TG/HDL	0.931
AIP	0.837
CRI-i=tc/HDL	0.796
CRI-II=LDL/HDL	0.683
AC	0.014
TCBI	0.051
Insulin resistance markers	
HOMA-IR	0.011
TyG	0.875
Tyg-BMI	0.450
TyG-WC	0.783
TyG-WHtR	0.591
Adiposity indices	
ABSI	0.145
TMI	0.843
ConI	0.113
VAI	0.063

*Note:* ABSI, Body Shape Index, ConI, Conicity Index; TyG, Triglyceride-Glucose Index; WHtR, waist-to-height ratio; TMI, Tri-Ponderal Mass Index.

Abbreviations: AC, atherogenic coefficient; AIP, Atherogenic Index of Plasma; CRI-I, Cardiometabolic Risk Index I; CRI-II, Cardiometabolic Risk Index II; TC, Total cholesterol; TCBI, Triglyceride-Cholesterol Body Index; TG, triglycerides; VAI, Visceral Adiposity Index.

**Table 4:** Summary of the principal component analysis (PCA) for atherogenic markers.

Principal component	Eigenvalue	Percentage of variance (%)	Cumulative percent of explainable variation (%)
PC1	5.47	45.55	45.55
PC2	2.53	21.06	66.61
PC3	1.45	12.11	78.72
Insulin resistance markers PCA			
PC1	3.28	46.80	46.80
PC2	1.22	17.40	64.20
PC3	0.86	12.23	76.43
Adiposity indices PCA			
PC1	2.26	37.69	37.69
PC2	1.23	20.44	58.13
PC3	1.07	17.88	76.00

## Data Availability

The data that support the findings of this study are available from the corresponding author upon reasonable request.

## Supporting Information

Supporting Information 1 Supporting Information Figure 1. Violin plot for atherogenic markers with subjects divided by sex, Figure S2.

Supporting Information 2 Supporting Information 2. Violin plot for atherogenic markers with subjects divided by vitamin D level.

Supporting Information 3 Supporting Information 3. Biplot graphs for atherogenic markers with comparison by sex.
