# Supplementary figures and images for: Exploring the Interplay Between Vitamin D and Metabolic Parameters in Pediatric Obesity: Toward Early Biomarker Detection
Source: J Nutr Metab. 2025 Nov 30;2025:4055705. doi: 10.1155/jnme/4055705 (PMC12682453; doi:10.1155/jnme/4055705)

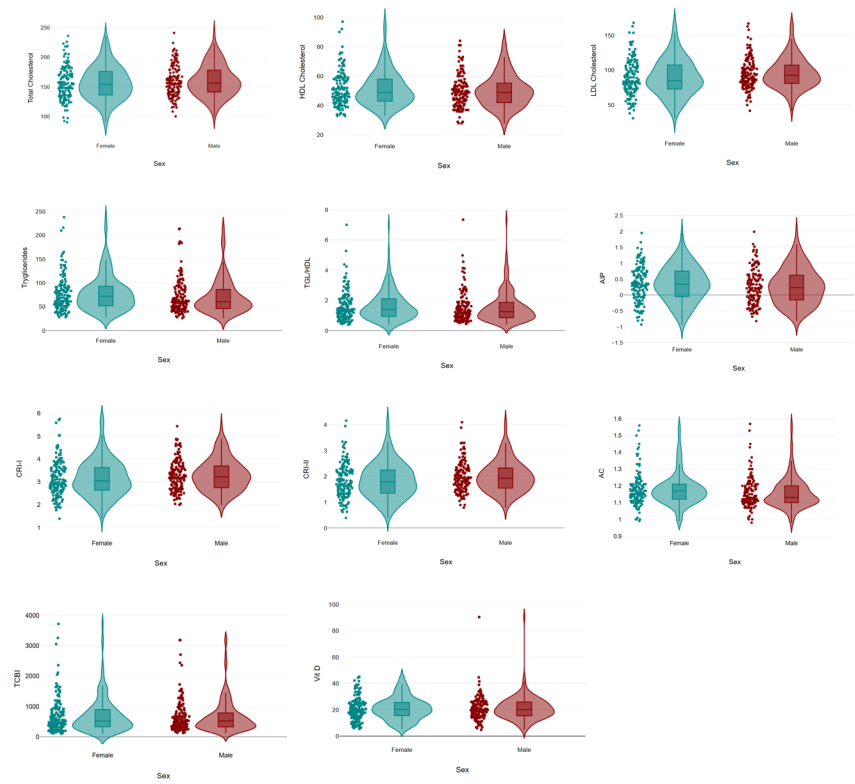

Supplement: Supporting Information 1 — Supporting Information Figure 1. Violin plot for atherogenic markers with subjects divided by sex, Figure S2. [file 4055705.f1.pdf]

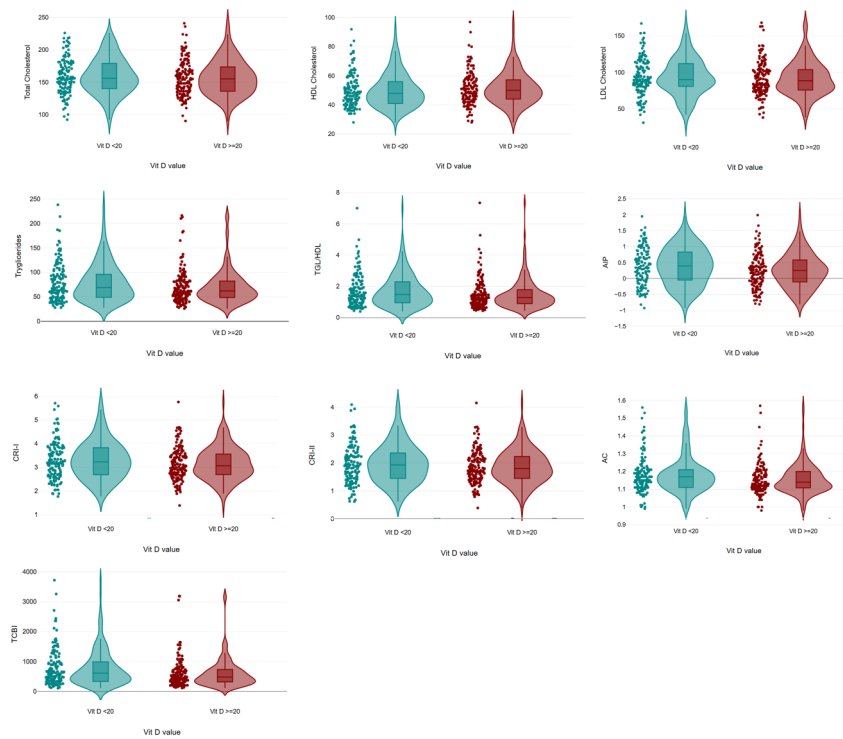

Supplement: Supporting Information 2 — Violin plot for atherogenic markers with subjects divided by vitamin D level. [file 4055705.f2.pdf]

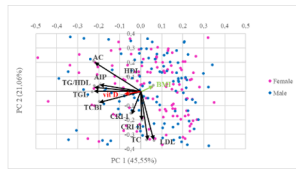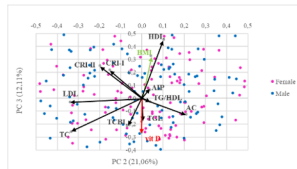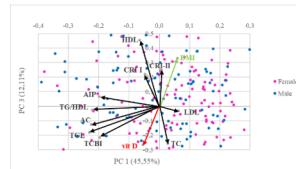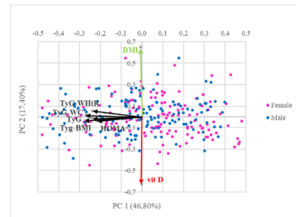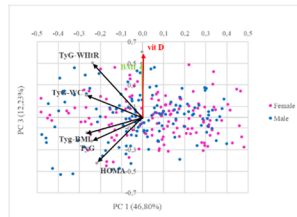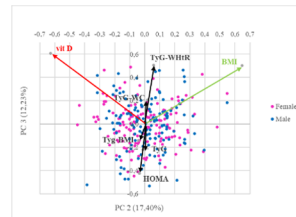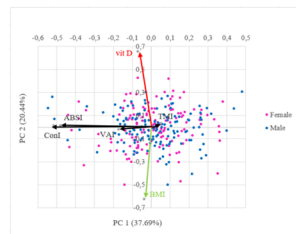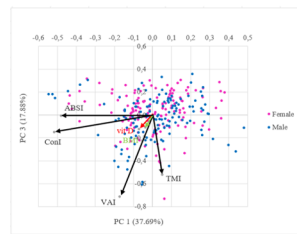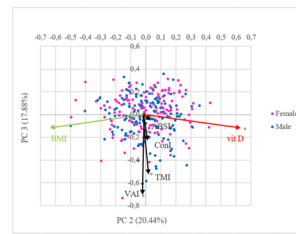

Supplement: Supporting Information 3 — Biplot graphs for atherogenic markers with comparison by sex. [file 4055705.f3.pdf]
